# Foretinib (GSK1363089) induces p53-dependent apoptosis in endometrial cancer

**DOI:** 10.18632/oncotarget.25232

**Published:** 2018-04-27

**Authors:** Yuhei Kogata, Tomohito Tanaka, Yoshihiro J. Ono, Masami Hayashi, Yoshito Terai, Masahide Ohmichi

**Affiliations:** ^1^ Department of Obstetrics and Gynecology, Osaka Medical College, Takatsuki, Japan

**Keywords:** endometrial cancer, foretinib, p53, apoptosis

## Abstract

**Objective:**

Foretinib (GSK1363089 or XL880), which is an oral multikinase inhibitor developed to primarily target the hepatocyte growth factor (HGF)/Met signaling pathway, has shown anti-tumor effects against some cancers in preclinical and clinical studies.

**Results:**

HGF/Met signaling in endometrial cancer cell lines was stimulated in an autocrine manner, and was essential for cell survival. Inhibiting the HGF/Met signaling with foretinib induced p53-dependent apoptosis in endometrial cancer cell lines *in vitro*. Foretinib also showed significant anti-cancer effects *in vivo* in experiments using cell tumor xenografts. p53 mutations were observed in 37 (10.8%) of 344 endometrial cancer specimens.

**Conclusion:**

The HGF/Met-MAPK/PI3K pathway in endometrial cancer is activated by HGF in an autocrine manner. Foretinib induces an anti-cancer effect through the anti-phosphorylation of Met, which results in the induction of p53-dependent apoptosis; foretinib was found to exert greater anti-cancer activity in endometrial cancer specimens with wild-type p53 than in specimens with p53 mutations. Our immunochemical analysis revealed that foretinib-induced p53-dependent apoptosis can be expected to have therapeutic potential in approximately 90% of endometrial cancer patients.

**Methods:**

We evaluated the HGF/Met signaling pathway in endometrial cancer cell lines and assessed the anti-cancer effects of foretinib using *in vitro* and *in vivo* experimental models. Furthermore, endometrial cancer specimens were subjected to an immunohistochemical analysis.

## INTRODUCTION

It has been estimated that more than sixty thousand women will be newly diagnosed with uterine corpus cancer, and that this cancer will be the cause of death in over ten thousand women in the United States in 2016 [1]. Endometrial carcinoma is the sixth most common cancer in women, and three-quarters of women who are diagnosed with endometrial cancer are postmenopausal. Approximately 80% of endometrial carcinomas are type I tumors, which are endometrioid carcinomas. Type I tumors are often preceded by endometrial hyperplasia, and are associated with estrogenic stimulation. Approximately 20% of endometrial carcinomas are type II tumors, which are nonendometrioid and largely serous carcinomas. Type II tumors arise in endometrial polyps or from endometrial intraepithelial carcinoma [2, 3]. Advanced endometrial carcinoma has basically been treated with radiation therapy as postoperative management; however, in recent years, radiation therapy has been compared to postoperative adjuvant chemotherapy and clinical trials of chemotherapy regimens have been conducted. However, the prognosis of advanced endometrial carcinoma is still poor, and molecular targeted therapy is attracting attention as a new treatment approach [3–5].

Tyrosine kinases, such as the receptors for epidermal growth factor (EGF), vascular endothelial growth factor (VEGF) and hepatocyte growth factor (HGF), are enzymes that selectively phosphorylate tyrosine residue in different substrates. Numerous researchers believe that the activation of tyrosine kinases promote tumorigenesis [6]. The overexpression of Met, the receptor for HGF, has been reported in many cancers of epithelial cell origin, including endometrial cancer [7]. After the binding of HGF, Met undergoes dimerization and autophosphorylation at specific tyrosine residues within the cytoplasmic domain, creating docking sites for intracellular signal transducers that activate Ras/mitogen-activated protein kinase (MAPK) and phosphatidylinositol 3-kinase (PI3K) [8, 9]. In several epithelial and mesenchymal cancers, the high expression of the Met protein has been reported to be an independent predictor of an adverse outcome [10–14], and Met was reported to activate cancer cell dissemination [14]. Consequently, HGF/Met signaling has become an attractive therapeutic target in cancer and has been the focus of numerous research studies. Foretinib (GSK1363089 or XL880) is an oral multikinase inhibitor that was developed to primarily target the signaling of HGF/Met and VEGF receptor-2 (VEGFR2) by binding to their adenosine triphosphate pockets [15]. In preclinical studies, foretinib reduced the overall tumor burden and metastasis in a xenograft mouse model of human ovarian cancer [16]. In a phase II trial of single-agent foretinib in patients with recurrent or metastatic squamous cell carcinoma, prolonged disease stabilization and a tolerable side-effect profile were observed [17]. In a phase I study of single-agent foretinib in patients with solid tumors, it was shown that foretinib inhibited Met phosphorylation and decreased the proliferation of tumors after treatment [18].

In a previous report, we investigated the HGF/Met signaling pathway in endometrial cancer cell lines [9]. In the report described above, we found that the levels of Met, Akt, and ERK phosphorylation were significantly decreased by foretinib treatment, suggesting that the PI3K/Akt and MAPK pathways are promoted via the HGF/Met signaling pathway in endometrial cancer cell lines. In the present study, we evaluated the HGF, which is activated in an autocrine manner, and the anti-cancer effects of foretinib using *in vitro* and *in vivo* experimental models.

## RESULTS

### Foretinib suppresses cell proliferation and induces apoptosis in endometrial cancer cell lines

**Figure 1:**
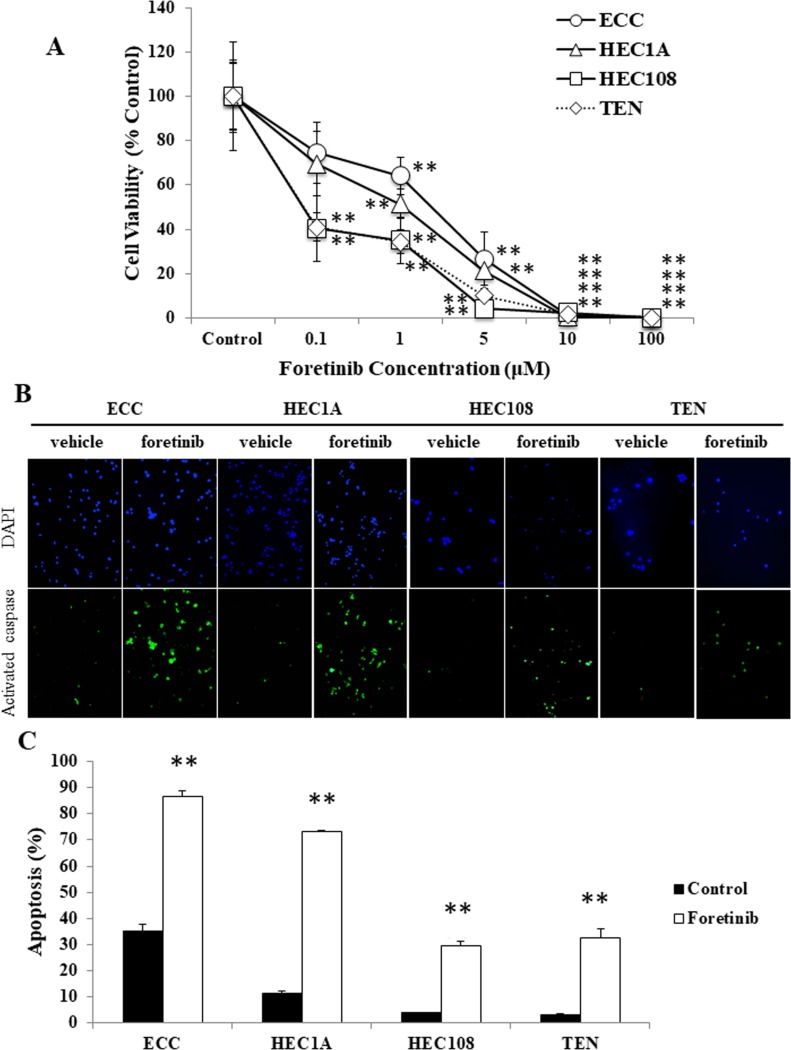
Foretinib induces apoptosis in endometrial cancer cell lines (**A**) EC cells were treated with various concentrations of foretinib for 48 hours and then their proliferation was measured by an MTS assay. (**B**, **C**) EC cells were treated with 3 μM foretinib for 48 hours and apoptotic cells were assessed by a caspase-3 activity assay. (B) Representative examples of immunostaining are shown on the left. FITC-labeled cells indicate the presence of apoptosis, and DAPI was used for counterstaining. (C) The percentage of apoptotic cells was counted using a FACScan device. Significant differences are indicated by asterisks. ^*^P < 0.05, ^**^P < 0.01.

To determine whether foretinib has any effect on the proliferation of EC cell lines, a MTS assay was performed using various concentrations of foretinib (0.1, 1, 5, 10, 100 μM). As shown in Figure 1A, the growth of the ECC, HEC-1A, HEC-108 and TEN cells was suppressed by foretinib in a concentration-dependent manner.

Next, to assess whether the suppression of cellular proliferation by foretinib was involved in the induction of apoptosis, an activated caspase-3 *in situ* detection assay was performed using vehicle (PBS) and 3 μM foretinib-treated EC cells. As shown in Figure 1B and 1C, the foretinib-treated cells exhibited significantly more apoptosis in comparison to the vehicle-treated cells in all cell lines.

### Met and Akt are phosphorylated in a sustained manner in endometrial cancer cell lines

**Figure 2:**
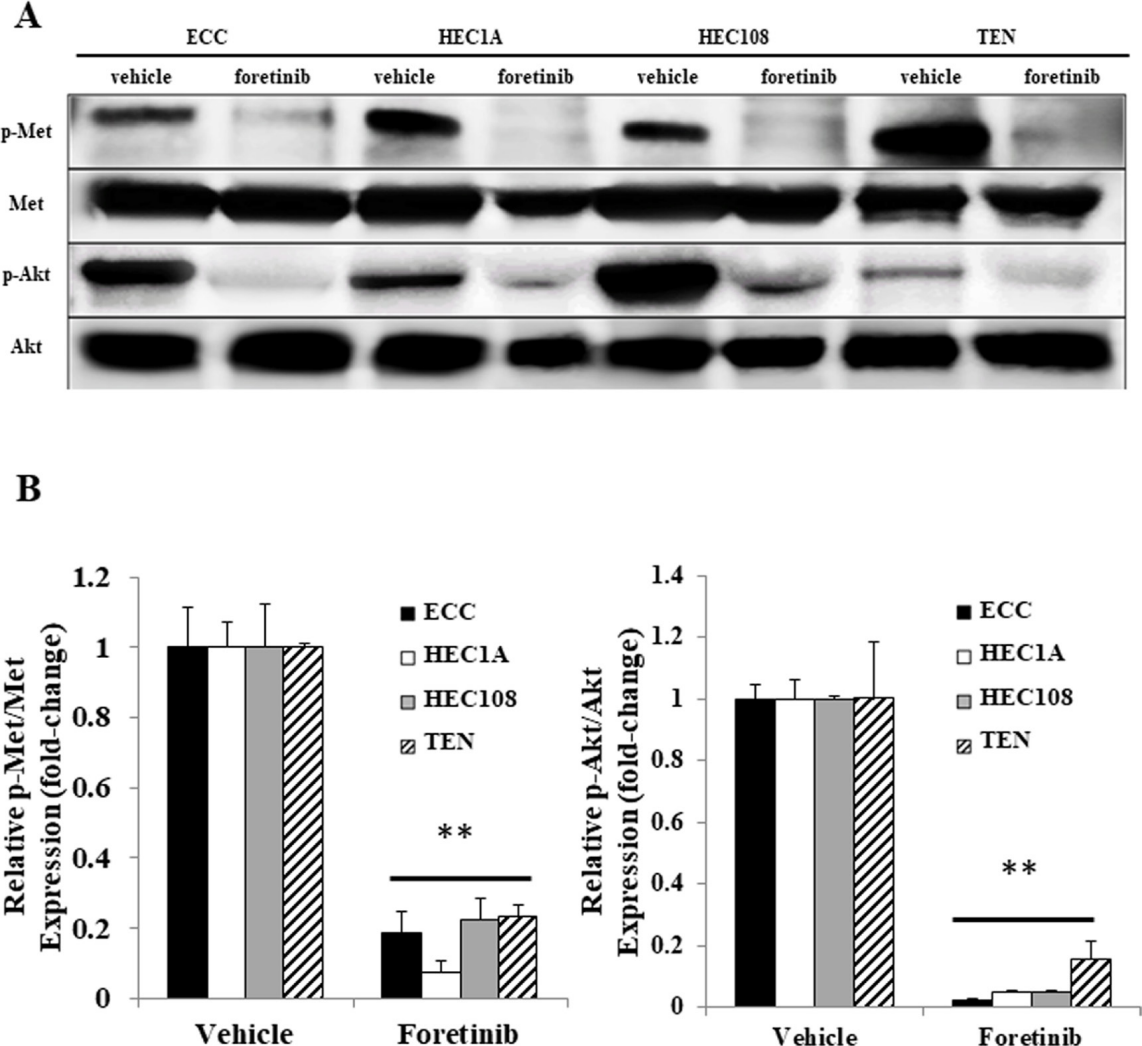
The Met and Akt phosphorylation in the endometrial cancer cell lines After serum starvation, the cells were treated with vehicle or foretinib for 24 hours. The cell lysates were analyzed by Western blotting using antibodies against p-Met, Met, p-Akt and Akt. Representative examples of bands from Western blots (**A**) and the densitometric quantification of the bands expressed as a fold-increase relative to the vehicle-treated cells (**B**). Significant differences are indicated by asterisks. ^**^P < 0.01.

Because foretinib induced basal cell apoptosis, we suspected that Met might be constantly phosphorylated in EC cell lines. The phosphorylation status of Met in EC cell lines was assessed by Western blotting. As shown in Figure 2, Met was constitutively phosphorylated in all four cell lines, and the addition of foretinib decreased the Met phosphorylation in all four cell lines. Since the PI3 kinase-Akt (PI3K) pathway has been reported to be activated by HGF/Met signaling [8, 9], the phosphorylation level of the Akt protein was also assessed by Western blotting. As shown in Figure 2, Akt was also constitutively phosphorylated in all four EC cell lines. Interestingly, Akt phosphorylation was similarly decreased by the addition of foretinib. These data indicate that the Met-Akt signaling is constitutively phosphorylated in EC cell lines.

### Foretinib induces p53-related apoptosis in EC cells

**Figure 3:**
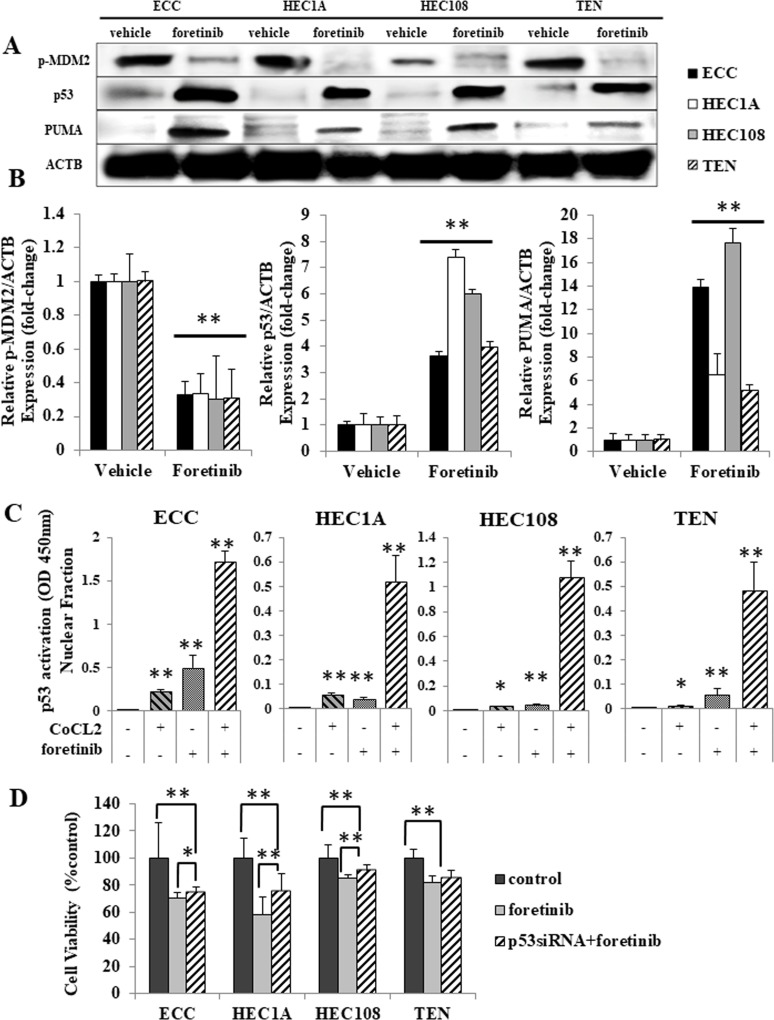
Foretinib induced p53-related apoptosis in endometrial cancer cell lines (**A**, **B**) After serum starvation, the cells were treated with vehicle or foretinib for 24 hours. The cell lysates were analyzed by Western blotting using antibodies against p-MDM2, p53, PUMA and ACTB. (A) Representative examples of bands from Western blotting (B); the densitometric quantification of the bands is expressed as the fold-increase relative to the vehicle treated cells. (**C**) Nuclear lysates were isolated and analyzed using a p53 Transcription Factor Assay kit according to the manufacturer's protocol (Cayman Chemicals), and the findings are expressed as the fold-increase relative to the basal transcription level in the control. Significant differences are indicated by asterisks. (**D**) EC cells transfected with the scrambled siRNA or p53 siRNA were treated with 5 μM foretinib for 24 hours and their proliferation was measured by an MTS assay. ^*^p < 0.05, ^**^P < 0.01.

It has been well documented that the activation of PI3K signaling increases the translocation of the mouse double minute 2 homolog (MDM2) from the cytoplasm to the nucleus, allowing p53 to be degraded by proteasomes [19]. Because Akt was constitutively phosphorylated in all four EC cell lines, we hypothesized that MDM2 would also be phosphorylated, and that p53 would be rapidly degraded, leading to the suppression of p53 activity. In order to assess the changes in the p53 activity of the EC cells, we examined the expression of p-MDM2, p53 and p53 upregulated modulator of apoptosis (PUMA), which is also known to be involved in p53-dependent apoptosis, by Western blotting. As shown in Figure 3A and 3B, MDM2 was constitutively phosphorylated without artificial stimulation, and the inhibition of HGF/Met signaling by foretinib reduced its phosphorylation in all four cell lines. In contrast, the expression of p53 and PUMA was either low or not observed in the vehicle-treated cells, and treatment with foretinib significantly increased the p53 and PUMA expression in all four cell lines.

Next, to verify whether foretinib activates p53 in EC cell lines, a p53 transcription factor assay was performed using the nuclear fraction from cells treated with foretinib and/or CoCl2, a chemical used to induce artificial hypoxia. As shown in Figure 3C, all cell lines demonstrated significant increases in p53 activation through CoCl2 treatment alone, which indicates that p53 was functional in all of the cell lines. Foretinib treatment induced a significant increase in the p53 activity, particularly in cells that were co-treated with CoCl2. These results suggest that foretinib induces and enhances the activation of p53 in EC cells.

Moreover, an MTS assay was performed to evaluate whether foretinib has a p53-dependent effect in p53 siRNA-transfected cells. Figure 3D shows that the anti-proliferation effect of foretinib was reduced in p53 siRNA-transfected cells.

### Met is phosphorylated in a sustained manner via autocrine HGF/Met signaling in EC cells

**Figure 4:**
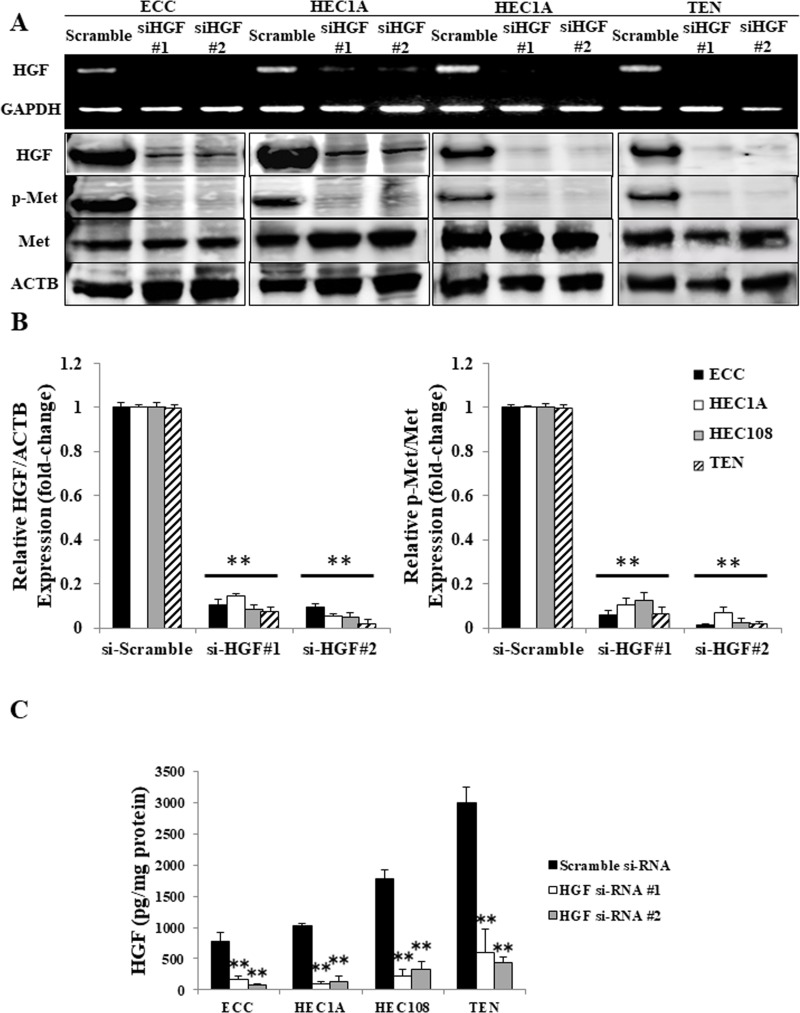
Autocrine HGF/Met signaling in EC cell lines (**A**) The mRNA expression of HGF and GAPDH was determined by an RT-PCR using total RNA, and the cell lysates were analyzed by Western blotting using antibodies against HGF, p-Met, Met and ACTB in the EC cells transfected with scrambled siRNA or HGF#1 or HGF#2 siRNA. Representative examples of bands from the RT-PCR (top) and Western blotting analyses (bottom). (**B**) The relative HGF/ACTB and p-Met/Met expression of the densitometric quantification of the bands from Western blotting expressed as the fold-increase relative to the basal transcription level in the scrambled siRNA-transfected cells. (**C**) The concentrations of HGF produced by EC cells transfected with the scrambled siRNA or HGF#1 or HGF#2 siRNA were measured using a Human HGF Quantikine ELISA Kit. Significant differences are indicated by asterisks. ^**^P < 0.01.

To confirm that autocrine HGF/Met signaling was the reason for the constant Met phosphorylation in EC cells, we performed experiments using HGF siRNA-transfected cells. As shown in Figure 4, the transfection of both the #1 and #2 HGF siRNAs apparently reduced the mRNA and protein levels of HGF (Figure 4A and 4B), as well as the secretion of HGF (Figure 4C) in all four cell lines. Moreover, as a result of the reduced HGF production and secretion, Met phosphorylation was also reduced (Figure 4A and 4B), confirming that the Met phosphorylation in EC cells occurs in an autocrine manner.

### Inhibiting autocrine HGF/Met signaling induces p53-related apoptosis in endometrial cancer cell lines

**Figure 5:**
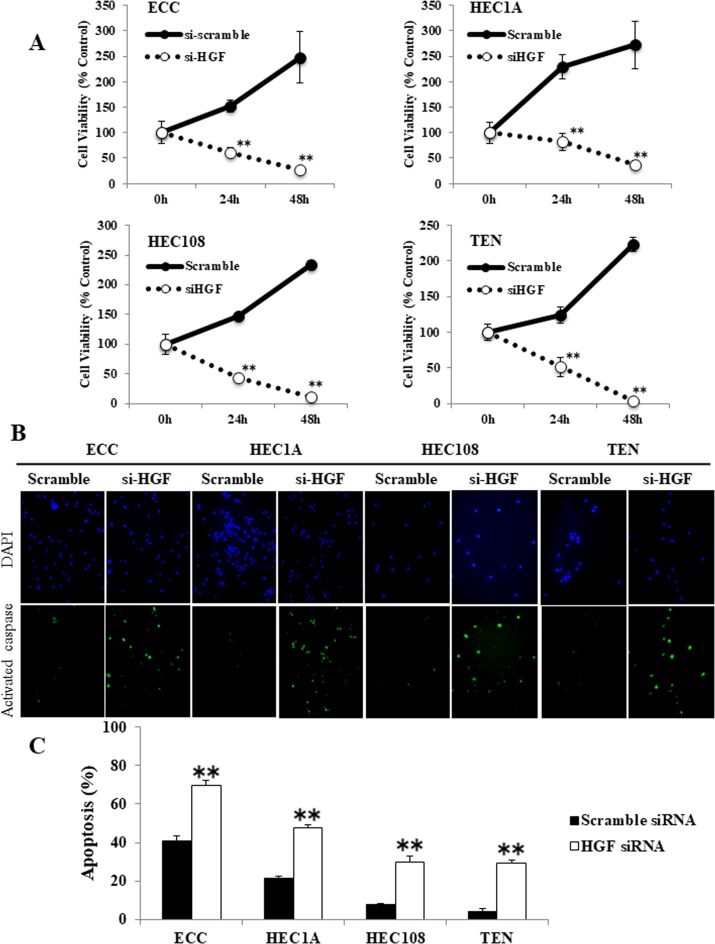
The inhibition of autocrine HGF/Met signaling induced apoptosis in endometrial cancer cell lines (**A**) EC cells were transfected with scrambled or HGF siRNA for 24 or 48 hours, then the cellular proliferation was measured by an MTS assay. (**B**, **C**) Apoptotic cells were assessed by a caspase-3 activity assay. (B) Representative examples of immunostaining are shown on the left. FITC-labeled cells indicated the presence of apoptosis, and DAPI was used for counterstaining. (C) The percentage of apoptotic cells was counted using a FACScan device. Significant differences are indicated by asterisks. ^**^P < 0.01.

Because foretinib is a receptor tyrosine kinase inhibitor that has inhibitory activity against both Met and VEGFR2, it was considered possible that the foretinib-induced apoptosis was the result of VEGFR2 inhibition and not Met inhibition. To examine this possibility, we assessed the proliferative and apoptotic status of HGF siRNA-transfected EC cells using an MTS assay and an activated caspase-3 *in situ* detection assay. As shown in Figure 5A, the HGF siRNA-transfected EC cells showed significant growth inhibition, which occurred in a time-dependent manner, in comparison to the scrambled siRNA-transfected cells. Moreover, HGF siRNA transfection induced a significantly greater level of apoptosis in comparison to the scrambled siRNA-transfected cells (Figure 5B and 5C). The fact that inhibiting autocrine HGF/Met signaling using HGF siRNA induced significant apoptosis in EC cell lines indicates that the inhibition of Met plays a large part in the growth effects in these four EC cell lines.

**Figure 6:**
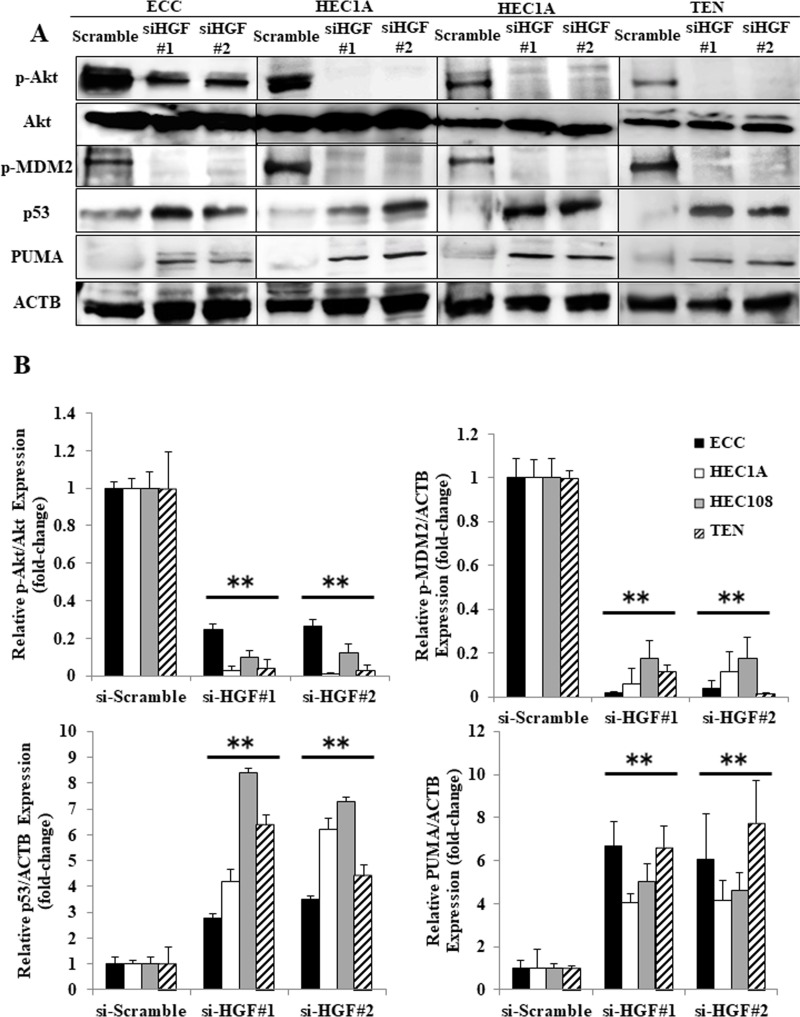
The inhibition of autocrine HGF/Met signaling induced p53-related apoptosis in endometrial cancer cell lines The cell lysates obtained from the EC cells that were transfected with the scrambled siRNA or HGF#1 or HGF#2 siRNA were analyzed by Western blotting using antibodies against p-Akt, Akt, p-MDM2, p53, PUMA and ACTB. Representative examples of the bands of the Western blots (**A**), and the densitometric quantification of the bands from a Western blot analysis that was normalized to the ACTB expression and expressed as the fold-increase relative to the basal transcription level in the scrambled siRNA-transfected cells (**B**). Significant differences are indicated by asterisks. ^**^P < 0.01.

In order to assess whether the p53-dependent apoptosis was induced via the inhibition of autocrine HGF/Met signaling, we examined the expression of p-Akt, p-MDM2, p53 and PUMA in HGF siRNA-transfected EC cells by Western blotting. As shown in Figure 6A and 6B, HGF siRNA transfection had similar results to foretinib treatment in EC cells (shown in Figures 2 and 3). Akt and MDM2 were phosphorylated and p53 was degraded in scrambled siRNA-transfected EC cells. In contrast, Akt and MDM2 phosphorylation were decreased, and the expression levels of p53 and PUMA were increased in HGF siRNA-transfected EC cells. These data suggest that autocrine HGF/Met signaling interferes with the p53 activity in EC cells, and that inhibiting autocrine HGF/Met signaling restores the p53 function.

### Foretinib induces p53-dependent apoptosis in EC tumor xenografts

**Figure 7:**
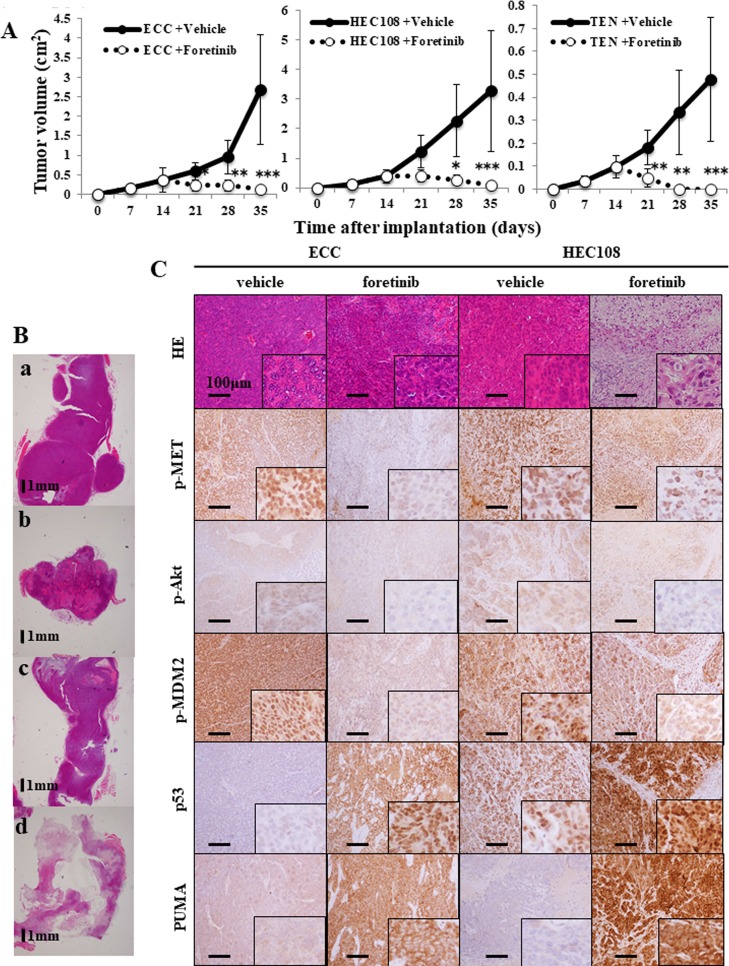
Foretinib treatment inhibits the growth of endometrial cancer cell xenografts (**A**) The growth of ECC, HEC-108 and TEN tumor xenografts in nude mice treated with foretinib or vehicle (1% saline). Treatment was started on day 14 post-tumor cell injection. The tumor diameters were measured using calipers and converted to volumes using the equation 1/2 (Length × Width^2^), where “Length” represents the larger diameter and “Width” represents the smaller diameter, respectively. Measurements were taken every week until day 35, when the animals were sacrificed. Significant differences are indicated by asterisks (vehicle-treated: ECC, *N* = 6; HEC-108, *N* = 6; TEN, *N* = 6 and foretinib-treated: ECC, *N* = 6; HEC-108, *N* = 6; TEN, *N* = 6). ^*^P < 0.05, ^**^P < 0.01, ^***^P < 0.001. (**B**, **C**) Xenograft tumors were fixed, embedded and sectioned. 7B-a: vehicle-treated ECC, b: foretinib-treated ECC, c: vehicle-treated HEC-108, d: foretinib-treated HEC-108. Immunohistochemical staining of the tumors for p-Met, p-Akt, p-MDM2, p53, PUMA and hematoxylin and eosin (HE) was performed. Representative histological images of the tumor xenografts are shown. Scale bars = 1 mm (B), 100 μm (C).

We evaluated the effects of foretinib on the growth kinetics of established ECC, HEC-108 and TEN cell tumor xenografts. The results showed that the daily oral administration of foretinib (30 mg/kg) significantly inhibited the growth of tumor in all three of the tumor xenografts starting after only seven days of administration, and lasting throughout the experiment (Figure 7A). Moreover, TEN cell tumor xenografts completely vanished in all specimens after 14 days of foretinib treatment. In order to evaluate the degree of apoptosis, we performed a histological analysis of the tumor. H&E staining showed an obvious increase in the necrotic tissue in the foretinib-treated tumors (Figure 7B). An immunohistochemical analysis showed an obvious increase in staining for cleaved caspase 3, PUMA and p53, and an obvious decrease in the staining for p-MDM2 in the foretinib-treated tumors in ECC and HEC-108 cell tumor xenografts, indicating that foretinib induced apoptosis and p53 activation in ECC and HEC-108 cell tumor xenografts. It was not possible to perform an immunohistochemical analysis for TEN cell tumor xenografts because the tumors vanished (Figure 7C).

### The immunochemical analysis of clinical specimens

Table 1 shows the characteristics of 344 Japanese patients with endometrial cancer. The mean (± SD) age of the patients was 58.0 ± 11.0 years. The mean body mass index was 24.1 ± 4.5 kg/m^2^. The FIGO classifications of the patients were as follows: stage I (*N* = 214; 62.2%), stage II (*N* = 20; 5.8%), stage III (*N* = 72; 20.9%) and stage IV (*N* = 21; 6.1%). The histological findings were as follows: atypical endometrial hyperplasia (*N* = 17; 4.9%), grade 1 or 2 endometrioid carcinoma (*N* = 231; 67.2%), grade 3 endometrioid carcinoma (*N* = 45; 13.1%), serous carcinoma (*N* = 18; 5.2%), clear cell carcinoma (*N* = 11; 3.2%), and carcinosarcoma (*N* = 22; 6.4%). Table 2 shows the association between the malignant potential of the tumor and the p53 status (determined by immunochemical staining). The findings were as follows: low-grade tumors including AEH and grade 1 or 2 endometrioid carcinoma (*N* = 248; 72.1%), high-grade tumors including grade 3 endometrioid carcinoma, serous carcinoma, clear cell carcinoma and carcinosarcoma (*N* = 96; 37.9%). Thirty-seven (10.8%) patients were positive for p53 and 307 (89.2%) were negative for p53. The rate of p53 positivity in the high-grade tumor group (24.0%) was significantly higher than that in the low-grade tumor group (6.0%). The rates of p53 positivity according were as follows: AEH (0%), G1 endometrioid carcinoma (5.2%), G2 endometrioid carcinoma (10.3%), G3 endometrioid carcinoma (26.7%), clear cell carcinoma (27.3%), serous carcinoma (16.7%) and carcinosarcoma (18.2%).

**Table 1:** The characteristics of the endometrial cancer patients

Number of patients	344 (%)
Age^*^	58.0 ± 11.0
BMI^*^	24.1 ± 4.5
FIGO stage	
I	214 (62.2)
II	20 (5.8)
III	72 (20.9)
IV	21 (6.1)
Histological type	
AEH	17 (4.9)
endometrioid G1 or G2	231 (67.2)
endometrioid G3	45 (13.1)
serous	18 (5.2)
clear cell	11 (3.2)
carcinosarcoma	22 (6.4)

^*^According to an ANOVA (mean ± SD); BMI, body mass index; AEH, atypical endometrial hyperplasia.

**Table 2:** The immunohistochemical analysis of the patients endometrial cancer patients

	Total	p53	
		positive (%)	negative (%)
Number of patients	344	37 (10.8)	307 (89.2)
Tumor Grade			
Type 1 (%)	248 (72.1)	15 (6.0)	233 (94.0)
AEH	17	0	17
endometrioid G1	173	9 (5.2)	164 (94.8)
endometrioid G2	58	6 (10.3)	52 (89.7)
Type 2 (%)	96 (37.9)	23 (24.0)	73 (76.0)
endometrioid G3	45	12 (26.7)	33 (73.3)
clear cell	11	3 (27.3)	8 (72.7)
serous	18	3 (16.7)	15 (83.3)
carcinosarcoma	22	4 (18.2)	18 (81.8)

AEH, atypical endometrial hyperplasia.

## DISCUSSION

In the present study, the HGF/Met-MAPK/PI3K pathway in endometrial cancer cell lines was activated by HGF in an autocrine manner. Foretinib was found to induce anti-cancer effects by the anti-phosphorylation of Met, which results in the induction of p53-dependent apoptosis; foretinib had more anti-cancer activity in endometrial cancer specimens with wild-type p53 than in specimens with p53 mutations. Our immunochemical analysis revealed that foretinib-induced p53 dependent apoptosis might have therapeutic potential in approximately 90% of patients with endometrial cancer.Although foretinib has shown anti-tumor activity in various cancers (*i.e.,* head and neck squamous cell cancer, hepatocellular carcinoma, papillary renal-cell carcinoma, gastric cancer, breast cancer and non-small cell lung cancer) in preclinical and clinical studies [20], because of its nature as an inhibitor of growth factors, it was believed that foretinib's mechanism of action merely involved suppressing the progression and spread of cancer, without a direct effect on cancer cell survival. However, in some preclinical studies, foretinib induced tumor hemorrhage and necrosis [20], suggesting that there was more to its activity than the mere suppression of cancer progression. HGF/Met signaling is not only a powerful stimulator of the invasion and metastasis of cells, but also allows for the survival of cancer cells in the bloodstream in absence of anchorage [21]. We herein demonstrated that the HGF/Met signaling in EC cell lines was stimulated in an autocrine manner, and that it was essential for cell survival. Given the importance of HGF/Met signaling in these cell lines, and the fact that foretinib is a kinase inhibitor designed to target HGF/Met signaling, the powerful anti-cancer effects of foretinib on EC cells that were observed in the current study are not surprising.

p53 is a transcription factor and inhibits cell proliferation by blocking the progression of the cell cycle and promoting apoptotic cell death; it thereby inhibits cancer development and regulates metabolism. Although the full spectrum of activity is still being uncovered, mutant p53 has been found in various cancers and involvement in the development and progression of cancer has been clarified [22, 23]. Mutant p53 has been found in approximately 10–20% of uterine endometrioid carcinomas and >90% of uterine serous or clear cell carcinomas. In addition, there is a trend toward a higher frequency of p53 mutations with increasing histopathological grades in uterine endometrioid carcinomas [23]. Vousden *et al.* reported that mutant p53 was found in 43% of Grade 3 uterine endometrioid carcinomas, while it was not detected in Grade 1 endometrioid carcinomas [22]. It also reported that p53 mutations are detected in 24–36% of clear cell carcinomas [24–26]. Mutations in p53 are not a simple phenomenon because some tumors harbor mutations in p53 that allow the protein to retain its functions [22]. We revealed that p53, which promote apoptosis, was viable in EC cell lines and that its function was suppressed via autocrine HGF/Met signaling. The p53 activity could be restored by suppressing the autocrine HGF/Met signaling, which resulted in p53-dependent apoptosis. Surprisingly, although the TEN cell line was derived from an endometrial clear cell carcinoma, the p53 function seemed to be viable, and foretinib was as effective against these cells as against the ECC, HEC-1A and HEC-108 cells which were derived from uterine endometrioid carcinomas. In addition to inducing p53-related apoptosis, foretinib has been reported to induce anticancer effects through the VEGF receptors. In a previous clinical study, foretinib was shown to have anti-cancer activity in triple-negative breast cancer with the low amplification of Met; foretinib has exerts anti-cancer effects through the inhibition of several pathways, in addition to the HGF/Met pathway. Although we showed that foretinib induces p53-related apoptosis in a previous study, foretinib was also observed to have a slightly inferior effect in p53 siRNA-transfected EC cell lines. Thus, foretinib showed antitumor effect in the absence of p53-related apoptosis. This also suggests that foretinib would be effective for treating high-grade endometrial cancer, which causes p53 mutations. Foretinib treatment had significant effects in all of our *in vivo* experiments, and the most notable finding was that it led to the complete disappearance of the TEN cell tumor xenografts. This finding could be important for patients with uterine clear cell carcinoma because clear cell carcinoma is known to have a poor prognosis due to the high rate of drug resistance [27, 28].

Another advantage of foretinib is that it is an orally administered drug with a tolerable side-effect profile [16, 17, 20, 29–31]. The most common adverse events include fatigue, constipation and hypertension, which are readily manageable, and the most common foretinib-related laboratory abnormalities are elevated AST and ALT, which are asymptomatic.

In conclusion, foretinib had therapeutic potential against EC cell lines in which it induced p53-dependent apoptosis, especially in clear cell carcinoma TEN. Additional subsequent studies will be needed to confirm the present findings. There are currently few effective treatments for progressive endometrial cancers, which metastasize and recur, and for types that are associated with a poor prognosis (*i.e.,* clear cell carcinoma); thus new treatments are required. Based on the results of this study, the foretinib may have potential therapeutic applications in the treatment of endometrial carcinoma by foretinib and combined use with other targeted drugs due to the low rate of adverse events may be considered. The use of foretinib in the treatment of endometrial cancer can be expected to achieve better outcomes.

## MATERIALS AND METHODS

### Reagents and antibodies

The rabbit polyclonal anti-human Met, rabbit polyclonal anti-human phospho-Met, rabbit polyclonal anti-human Akt, rabbit polyclonal anti-human phospho-Akt, rabbit polyclonal anti-human p53, rabbit polyclonal anti-human phospho-MDM2, rabbit polyclonal anti-human PUMA and rabbit monoclonal anti-human β−actin antibodies that were used for immunoblotting and immunohistochemistry were all purchased from Cell Signaling Technology, Inc. (Danvers, MA, USA). The rabbit polyclonal anti-human HGF (ab24865) used for immunoblotting and immunohistochemistry was purchased from Abcam (Cambridge, MA, USA). The rabbit polyclonal anti-human acetyl-p53 (Lys320) antibody that was used for immunoblotting and the rabbit anti-human Ki-67 antibody that was used for immunohistochemistry were purchased from Merck Millipore (Billerica, MA, USA). Foretinib (GSK1363089) was purchased from Selleck Chemicals (Houston, TX, USA). Cobalt (II) chloride (CoCl2) was purchased from Sigma-Aldrich (St. Louis, MO, USA). The BD Falcon™ 96-well microplates that were used for the cell proliferation assays were purchased from BD (Franklin Lakes, NJ, USA).

### Cell lines and conditioned media

The human endometrioid adenocarcinoma cell lines (ECC, HEC-1A and HEC-108) were purchased from American Type Culture Collection (Rockville, MD) in 2013; TEN cells were purchased from RIKEN BRC through the National Bio-Resource Project of MEXT, Japan (Ibaraki, Japan). ATCC and RIKEN BRC routinely authenticate their cell lines by Short Tandem Repeat (STR) polymorphism profiling analyses. We also performed an STR polymorphism profiling analysis (Wakennyaku co., Ibaraki, Japan) to confirm the cell line identity. All of the experiments were performed at passages <15–20.

All cell lines were grown in culture dishes (Becton Drive, Franklin Lakes, NJ) in DMEM supplemented with 10% charcoal-stripped fetal bovine serum (FBS) (Equitech-Bio, Kerrville, TX) (growth medium) in an atmosphere of 5% CO_2_ at 37°C. Serum-free DMEM was used for cell starvation. The ECC, HEC-1A and HEC-108 cells were derived from well-differentiated, grade 2 and grade 3 endometrioid adenocarcinomas of the uterus, respectively. TEN cells were derived from an endometrial clear cell carcinoma.

### RNA extraction, and the semi-quantitative reverse transcription polymerase chain reaction (RT-PCR)

Total RNA was obtained from cultured cells using the RNeasy Mini kit (Qiagen, Germantown, MD, USA), and 2 μg of RNA was subsequently reverse-transcribed with Superscript II reverse transcriptase (Invitrogen, Carlsbad, CA, USA) using random primers according to the manufacturer's instructions. The cDNA was then amplified using Taq DNA polymerase (Roche Diagnostics, Mannheim, Germany). The primers used were as follows: *HGF*, forward: 5′-GCTTGCTCCTCCCTTCCTTAC-3′, and reverse: 5′-AGTTTGGTCACCCACATGGT-3′, *GAPDH*, forward: 5′-AGCCACATCGCTCAGACA-3′ and reverse: 5′-GCCCAATACGACCAAATCC-3′.

### Western blotting

Western blotting was performed as described previously [32]. Briefly, total proteins were prepared using Pierce RIPA Buffer (Thermo Fisher Scientific, MA, USA). Equal amounts of whole-cell proteins were separated via SDS polyacrylamide gel electrophoresis and electrotransferred to nitrocellulose membranes. Non-specific antigen sites were blocked with 10% bovine serum albumin in 1× Tris-buffered saline for 1 h. Western blotting was performed with various specific primary antibodies (described above). The immunoreactive bands in the immunoblots were visualized using horseradish peroxidase-coupled goat anti-rabbit immunoglobulin using an enhanced chemiluminescence Western blotting system (ECL Plus, GE Healthcare Life Sciences, Pittsburgh, PA, USA). All of the experiments were repeated at least three times and yielded similar results.

### Detection of apoptotic cells

The apoptosis of EC cells was assessed under various conditions by a caspase activity assay using the CspACETM FITC-VAD-FMK *In Situ* Marker (Promega, WI, USA), in accordance with the manufacturer's instructions. The slides were analyzed with a confocal laser scanning microscope (Carl Zeiss Microscopy GmbH, Gottingen, Germany), and a flow cytometry analysis was conducted using a FACScan system in order to quantify the level of apoptosis based on the rate of testosterone depletion. Approximately 10,000-gated events were analyzed per sample. The results were analyzed using the Windows Multiple Document Interface flow cytometry applications software program, and the rate of apoptosis was calculated.

### The enzyme-linked immunosorbent assay (ELISA)

The cells were cultured in six-well plates with growth media until they reached 70–80% confluence then were starved for 16 h. The culture supernatants were collected after another 48 h of incubation and were analyzed using an ELISA with a human HGF immunoassay (Abcam, Cambridge, MA) according to the manufacturer's instructions. The absorbance was read at 450 nm using a Corona SH-1000 lab absorbance microplate reader (Corona Electric Co. Inc, Ibaraki, Japan). The sample concentrations were determined via interpolation from the standard curve. Five independent experiments were performed, and the ratio was expressed as the mean ± SD.

### Small interfering RNA (siRNA) transfection

The siRNA specific for HGF, as well as scrambled control, was purchased from Invitrogen and siRNA specific to p53 was purchased from Santa Cruz Biotechnology (Dallas, TX, USA). Cells were transfected using Lipofectamine (Invitrogen) according to the manufacturer's instructions. Briefly, oligomer-Lipofectamine PLUS complexes were prepared as follows: 20 pmol siRNA oligomer was diluted in 50 μl of Opti-MEM (Invitrogen). Lipofectamine PLUS was mixed gently before use, and then a 1-μl aliquot was diluted in 50 μl of Opti-MEM, mixed gently, and incubated for 5 min at room temperature. After 5 min of incubation, the diluted oligomer was combined with the diluted Lipofectamine PLUS, mixed gently, and incubated for another 20 min at room temperature. The oligomer-Lipofectamine PLUS complexes were added to each well containing cells and medium, and were mixed gently by rocking the plate back and forth. The cells were incubated at 37°C in a CO_2_ incubator for 24 h, and were then prepared for each assay.

### The p53 transcript DNA-binding assay

Nuclear fractions were extracted using a Nuclear Extract Kit (Active Motif, CA, USA). Specific transcription factor DNA binding in the nuclear extracts post-treatments was detected using the sensitive, non-radioactive Cayman p53 Transcription Factor Assay kit (Cayman Chemical Company, MI, USA). The procedures used to prepare the nuclear extracts and to assess the transcriptional activity were performed according to the manufacturer's instructions.

### Immunohistochemistry

Tissue samples were formalin-fixed and embedded in paraffin. Deparaffinized and rehydrated sections (4 μm) were autoclaved in 0.01 mol/l citrate buffer (pH 6.0) for 15 min at 121°C for antigen retrieval. The endogenous peroxidase activity was blocked with 0.3% hydrogen peroxide solution in methanol for 30 min, then sections were incubated at 4°C for 12 h with anti-phosphorylated Akt antibody (1:100 dilution), anti-phosphorylated MET antibody (1:100 dilution), anti-phosphorylated MDM2 antibody (1:100 dilution), anti-p53 antibody (1:200 dilution), anti-PUMA antibody (1:500 dilution) or anti-cleaved caspase-3 antibody (1:800 dilution). The sections were then washed with 1× phosphate-buffered saline (PBS) and incubated with Histofine Simple Stain MAX PO (multi; Nichirei) for 30 min at room temperature. Finally, the sections were washed with 1× PBS and visualized by incubation with H_2_O_2_/diaminobenzidine substrate solution for 5 min. The sections were counterstained with hematoxylin prior to dehydration and mounting. Two independent pathologists who were blinded to the clinicopathological data evaluated the immunohistochemical data. Immunostaining intensity was classified into two categories: p53-positive (staining of >10% of the tumor) and p53-negative (staining of <10% of the tumor) [33].

### Xenograft study

After receiving approval from the Animal Care and Use Committee of Osaka Medical College, six-week-old female athymic nude mice (BALB/c Slc-nu/nu) were used for the xenograft study. All mice were purchased from Japan SLC, Int. and were housed three mice per cage. The mice had *ad libitum* access to sterile food pellets and water. All of the procedures were approved by the Osaka Medical College Animal Committee, and followed the institutional guidelines for animal welfare and experimental conduct. A total of 10^6^ ECC cells were into the upper left fat pad of 12 mice, while 10^6^ HEC-108 cells or 10^6^ TEN cells were injected into the upper right fat pad of 12 different mice. Foretinib was suspended in 1% saline (Sigma-Aldrich) at a concentration of 6 mg/ml, and the daily oral administration of foretinib (30 mg/kg) was started 14 days after cell injection (ECC: *N* = 6, HEC-108: *N* = 6, TEN: *N* = 6); 50 μl of 1% saline was orally administered every day to the vehicle group (ECC: *N* = 6, HEC-108: *N* = 6, TEN: *N* = 6). The tumor volumes were determined as described previously (Reed *et al.* 2005). All animals were sacrificed on day 35 or when the mean diameter of the tumor reached 1.5 cm, and the tumors, lungs, livers, and hearts were collected and snap-frozen or fixed.

### Patients and tissue samples

The present study included 344 Japanese patients with endometrial cancer who were treated at Osaka Medical College between 2002 and 2010. Patients who had undergone hysterectomy as an initial treatment and for whom sufficient clinical data were available regarding the oncologic outcome (including the date of recurrence) were eligible for inclusion in the present study. All patients were staged according to the International Federation of Gynecology and Obstetrics (FIGO) criteria. The histological subtype was assigned according to the criteria of the World Health Organization classification. Most patients who had deep half myometrial invasion or high-grade tumors received postoperative adjuvant therapy, which included chemotherapy or pelvic irradiation. The present study was approved by the institutional review board (IRB) of Osaka Medical College. Written informed consent was obtained from some patients for the use of their tissue samples and the clinical records in the present study. The other patients provided their written informed consent for the use of their tissue samples and clinical records for an IRB-approved study at the time of primary surgery; the consent procedure was approved by the IRB.

### Statistics

The statistical analyses were performed using the StatView software program (SAS Institute, Cary, NC), and the statistical significance of differences was determined using the Kruskal-Wallis and Mann-Whitney *U* test or a paired *t*-test, as appropriate. *P* values of < 0.05 were considered to indicate statistical significance.

## Abbreviations

EGF: epidermal growth factor
VEGF: vascular endothelial growth factor
HGF: hepatocyte growth factor
MAPK: mitogen-activated protein kinase
PI3K: phosphatidylinositol 3-kinase
VEGFR2: VEGF receptor-2
EC: endometrial cancer
PI3K: PI3 kinase-Akt
MDM2: mouse double minute 2 homolog
PUMA: p53 upregulated modulator of apoptosis
CoCl2: Cobalt (II) chloride
STR: Short Tandem Repeat
FBS: fetal bovine serum
RT-PCR: reverse transcription and semi-quantitative polymerase chain reaction (RT-PCR)
ELISA: Enzyme-linked immunosorbent assay
siRNA: Small interfering RNA.
